# Nitric oxide-induced eosinophil apoptosis is dependent on mitochondrial permeability transition (mPT), JNK and oxidative stress: apoptosis is preceded but not mediated by early mPT-dependent JNK activation

**DOI:** 10.1186/1465-9921-13-73

**Published:** 2012-08-24

**Authors:** Pinja Ilmarinen-Salo, Eeva Moilanen, Vuokko L Kinnula, Hannu Kankaanranta

**Affiliations:** 1 The Immunopharmacology Research Group, University of Tampere School of Medicine and Tampere University Hospital, Tampere, Finland; 2 Department of Medicine, Division of Pulmonary Medicine, University of Helsinki and Helsinki University Central Hospital, Helsinki, Finland; 3 Department of Respiratory Medicine, Seinäjoki Central Hospital, Seinäjoki, Finland

**Keywords:** Eosinophils, Apoptosis, Nitric oxide, Mitochondrial permeability transition, JNK, Reactive oxygen species

## Abstract

**Background:**

Eosinophils are critically involved in the pathogenesis of asthma. Nitric oxide (NO) is produced in high amounts in asthmatic lungs and has an important role as a regulator of lung inflammation. NO was previously shown to induce eosinophil apoptosis mediated via c-jun N-terminal kinase (JNK) and caspases. Our aim was to clarify the cascade of events leading to NO-induced apoptosis in granulocyte macrophage-colony stimulating factor (GM-CSF)-treated human eosinophils concentrating on the role of mitochondria, reactive oxygen species (ROS) and JNK.

**Methods:**

Apoptosis was determined by flow cytometric analysis of relative DNA content, by Annexin-V labelling and/or morphological analysis. Immunoblotting was used to study phospho-JNK (pJNK) expression. Mitochondrial membrane potential was assessed by JC-1-staining and mitochondrial permeability transition (mPT) by loading cells with calcein acetoxymethyl ester (AM) and CoCl_2_ after which flow cytometric analysis was conducted. Statistical significance was calculated by repeated measures analysis of variance (ANOVA) or paired t-test.

**Results:**

NO-donor S-nitroso-N-acetyl-D,L-penicillamine (SNAP) induced late apoptosis in GM-CSF-treated eosinophils. SNAP-induced apoptosis was suppressed by inhibitor of mPT bongkrekic acid (BA), inhibitor of JNK SP600125 and superoxide dismutase-mimetic AEOL 10150. Treatment with SNAP led to late loss of mitochondrial membrane potential. Additionally, we found that SNAP induces early partial mPT (1 h) that was followed by a strong increase in pJNK levels (2 h). Both events were prevented by BA. However, these events were not related to apoptosis because SNAP-induced apoptosis was prevented as efficiently when BA was added 16 h after SNAP. In addition to the early and strong rise, pJNK levels were less prominently increased at 20–30 h.

**Conclusions:**

Here we demonstrated that NO-induced eosinophil apoptosis is mediated via ROS, JNK and late mPT. Additionally, our results suggest that NO induces early transient mPT (flickerings) that leads to JNK activation but is not significant for apoptosis. Thereby, we showed some interesting early events in NO-stimulated eosinophils that may take place even if the threshold for irreversible mPT and apoptosis is not crossed. This study also revealed a previously unknown physiological function for transient mPT by showing that it may function as initiator of non-apoptotic JNK signalling.

## Introduction

Eosinophils play a crucial role in the pathogenesis of asthma. By releasing toxic granule proteins, lipid mediators and other proinflammatory components, eosinophils contribute especially to exacerbations of asthma [1]. Additionally, eosinophils have gained increasing attention as antigen-presenting cells and as important regulators of T-helper (Th) 2 cytokine production and airway remodelling [2,3]. Eosinophils typically exist in low numbers in human peripheral blood complicating studies on their functions. They undergo spontaneous apoptosis in few days in the absence of any survival-prolonging cytokines. Blood eosinophils obtained from patients with asthma show delayed apoptosis when compared to eosinophils from healthy controls [4] and elevated levels of eosinophil survival-prolonging cytokines interleukin (IL)-5 and granulocyte macrophage-colony stimulating factor (GM-CSF) have been found from the bronchoalveolar lavage fluid of asthmatics [5]. GM-CSF has been demonstrated as the main eosinophil-survival prolonging cytokine in asthmatic airways [6]. Apoptosis is an efficient way to discard eosinophils from the airways by avoiding inflammation. It is characterized by cell shrinkage, chromatin condensation, nuclear coalescence, DNA fragmentation, mitochondrial changes and transfer of phosphatidyl serine residues from the inner to the outer leaflet of the cell membrane.

Nitric oxide (NO) is a gaseous molecule possessing both physiological and pathophysiological role in human tissues [7]. During inflammation, inducible nitric oxide synthase (iNOS) is rapidly activated by bacterial endotoxin and inflammatory cytokines IL-1, tumor necrosis factor (TNF)-α and IFN-γ resulting in high production of NO. In inflamed airways, NO is produced by iNOS in alveolar macrophages and bronchial epithelial cells. Patients with asthma show elevated levels of NO in the exhaled air, which correlates to clinical symptoms of asthma, sputum eosinophilia and eosinophil activation markers [8,9]. However, in a rat-model of asthma NO-releasing glucocorticoid was shown to be more potent than glucocorticoid alone. In addition, NO-releasing compound diethylenetriamine (DETA)-NONOate was shown to be as potent as a glucocorticoid in inhibiting eosinophilic inflammation [10]. NO may have both pro- and anti-inflammatory properties in asthmatic inflammation.

Interestingly, we have previously shown that exogenous NO induces human eosinophil apoptosis *in vitro* in the absence and presence of IL-5 and GM-CSF, which may act as a counter regulatory mechanism to limit eosinophilia in inflamed lungs [11,12]. Apoptotic rate of sputum eosinophils was found to positively correlate with exhaled NO in children [13] indicating that induction of eosinophil apoptosis by NO may have clinical relevance. NO was shown to possess its pro-apoptotic effect via c-Jun-N-terminal kinase (JNK) [11] and caspases 6 and 3 [12]. In previous studies with other cell types and cell-free systems treatment with NO has been found to lead to formation of reactive oxygen species (ROS), stimulation of mitochondrial permeability transition (mPT) and disruption of mitochondrial function [14,15]. Mitochondrial permeability transition pore is a Ca^2+^- and voltage-dependent channel in mitochondrial inner membrane for molecules up to 1.5 kDa. Ca^2+^-overload induces mPT pore to open resulting in equilibration of small molecules across the inner membrane, loss of mitochondrial membrane potential (ΔΨ_m_), mitochondrial swelling and finally rupture of the outer mitochondrial membrane which releases cytochrome c and other pro-apoptotic factors to cytosol to initiate apoptosis [16]. Only scarce information exists of the function of mPT in eosinophils [17]. JNK is a stress-regulated kinase that has been previously shown to mediate apoptosis by increasing transcription of several pro-apoptotic molecules and by phosphorylating B-cell lymphoma (Bcl) 2 family members thereby participating in mitochondrial apoptotic pathway [18]. This study was conducted to find out the cascade of events and signalling mechanisms leading to NO-induced eosinophil apoptosis in the presence of survival-prolonging cytokine GM-CSF, especially concentrating on the role of ROS, JNK and mitochondria.

## Methods

### Materials

AEOL 10150 was a kind gift from Prof. James Crapo (University of Colorado, Denver, USA). Materials were purchased as previously described [12] or as follows: SP600125, negative control for SP600125, JNK inhibitor VIII, bongkrekic acid, apocynin (Merck, Darmstadt, Germany), diphenyleneiodonium chloride (DPI) (Sigma-Aldrich Co., St. Louis, MO, USA), JC-1 mitochondrial membrane potential detection kit (Biotium Inc., Hayward, CA, USA), MitoProbe transition pore assay kit (Molecular Probes Inc., Eugene, OR, USA), phospho-JNK (pJNK) antibody (Thr183/Tyr185) (Cell Signaling Technology Inc., Danvers, MA, USA), JNK antibody (Santa Cruz Biotechnology, Santa Cruz, CA, USA).

### Human eosinophil purification and culture

The blood samples (100 ml) were taken from healthy, allergic or asthmatic individuals. All donors gave written informed consent to a study protocol approved by the ethical committee of Tampere University Hospital. Eosinophils were isolated to >99% purity and cultured under sterile conditions as previously described [12].

### Apoptosis assays and western blotting

Relative DNA fragmentation assay of propidium iodide (PI)-stained cells, morphological analysis, Annexin-V binding assay, eosinophil lysis and western blotting were performed as previously described [12].

### Measurement of mitochondrial membrane potential

Mitochondrial membrane potential (ΔΨ_m_) was determined by staining eosinophils with cationic mitochondrial dye JC-1 followed by flow cytometric analysis [19]. In cells with intact ΔΨ_m_ JC-1 accumulates into mitochondrial matrix. When the critical concentration is achieved, JC-1 aggregates releasing red fluorescence. In cells with collapsed ΔΨ_m_ JC-1 remains in a monomeric form in the cytoplasm emitting green fluorescence. Cells (2.5-5 x 10^5^) were stained for 15 min with JC-1 at 37°C, washed twice in medium and re-suspended in PBS, after which flow cytometric analysis was conducted. K^+^ ionophore valinomycin is a drug able to collapse ΔΨ_m_ and was used as a positive control [19]. The gates defining cells with intact and lost ΔΨ_m_ were based on cells treated with GM-CSF (10 pM) and valinomycin (1 μM), respectively, in each experiment individually.

### Determination of mitochondrial permeability transition

Mitochondrial permeability transition was determined by MitoProbe transition pore assay kit (Molecular Probes Inc., Eugene, OR, USA), a technique based on calcein acetoxymethyl ester (AM) and CoCl_2_[20]. Calcein AM is a non-fluorescent molecule which accumulates into cytosol and mitochondria. Cleavage of calcein AM by intracellular esterases liberates fluorescent calcein dye that does not cross plasma or mitochondrial membrane. In normal cells, addition of CoCl_2_ quenches the fluorescence of cytosolic but not mitochondrial calcein. In cells with mPT, CoCl_2_ enters also mitochondria to quench the mitochondrial calcein fluorescence. Calcein AM/CoCl_2_-method requires activity of intracellular esterases that are functional only in vital cells restricting use of this method. Cells (5 x 10^5^) were labeled with 10 nM calcein AM, 400 μM CoCl_2_ and/or 0.5 μM ionomycin for 15 min at 37°C. Cells were washed with HBSS, resuspended in PBS and analyzed by flow cytometry. Ionomycin, known to induce complete mPT, always produced minimal fluorescence of 2–4 mean fluorescence intensity (MFI) in the presence or absence of GM-CSF, penicillamine or SNAP indicative of complete mPT. Calcein fluorescence is expressed as percentage of its initial value ((mean of calcein fluorescence of cells treated with calcein AM and CoCl_2_ / mean of calcein fluorescence of cells treated with calcein AM) * 100). This data was finally normalized against control cells (cells treated with GM-CSF and penicillamine).

### Statistics

Results are shown as mean ± SEM. Statistical significance was calculated by repeated measures analysis of variance with Dunnett’s post-test or by paired t-test by using GraphPad InStat version 3.05 (GraphPad Software, San Diego, CA, USA). Differences were considered significant when p < 0.05.

## Results

### SNAP induces apoptosis in GM-CSF-treated eosinophils

**Figure 1:**
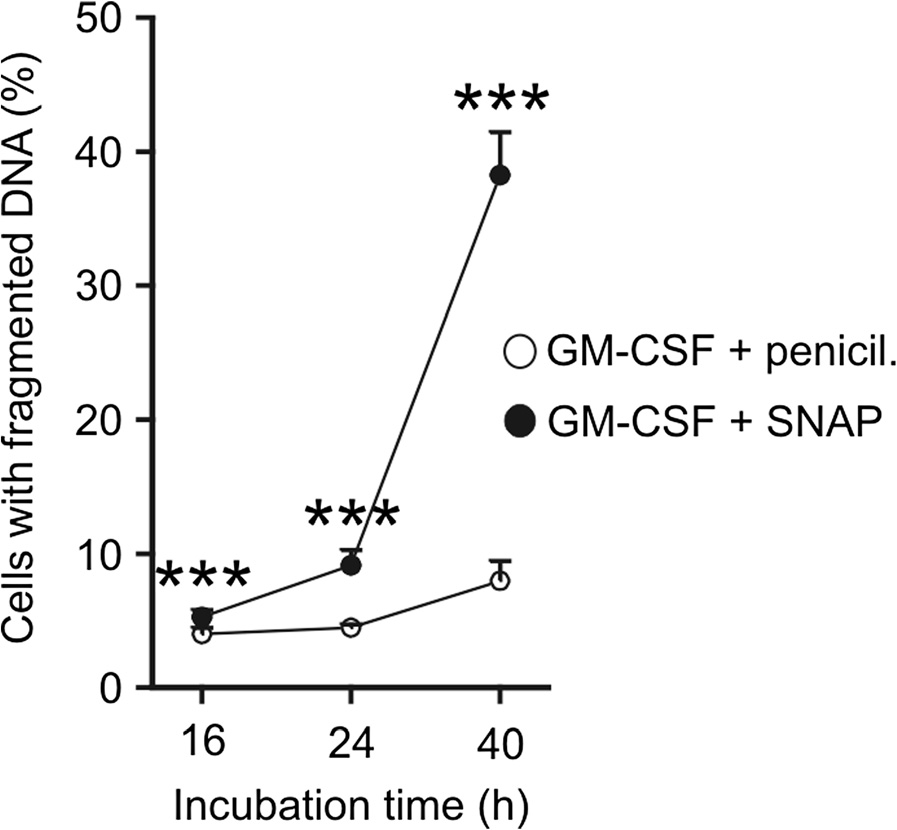
**SNAP induces late apoptosis in GM-CSF-treated eosinophils.** Eosinophils were incubated in the presence or absence of 1 mM SNAP, 1 mM penicillamine and/or 10 pM GM-CSF for the indicated times. Apoptosis was measured by DNA fragmentation assay. *** indicates p < 0.001 as compared with cells treated with GM-CSF and penicillamine at the corresponding time-point. Mean ± SEM is shown from six independent experiments.

NO-donor SNAP but not the negative control N-acetyl-D,L-penicillamine induced apoptosis in GM-CSF-treated eosinophils as previously described and as indicated in Table 1[12]. Time-course of SNAP-induced apoptosis revealed late initiation of cell death. SNAP induced only a minor increase in DNA fragmentation at 16 and 24 h time-points and a significant increase was observed only after 40 h of incubation (Figure 1).

**Table 1:** **Effect of SNAP on GM-CSF-induced eosinophil survival as previously described**[12]

	**Apoptotic cells (%)**
Untreated	60.8 ± 4.1
GM-CSF 10 pM	11.1 ± 1.6
+SNAP 10 μM	10.5 ± 1.8
+SNAP 100 μM	28.4 ± 10.9
+SNAP 1000 μM	44.6 ± 8.6 **
GM-CSF 10 pM	9.3 ± 1.0
+Penicillamine 100 μM	8.6 ± 1.2
+Penicillamine 1000 μM	8.6 ± 2.1

Apoptosis was determined after 40 h of incubation by DNA fragmentation assay. Shown are mean ± SEM, n = 7. ** indicates p < 0.01 as compared with GM-CSF-treated cells.

### SNAP-induced apoptosis is dependent on late mPT but preceded by early partial mPT

**Figure 2:**
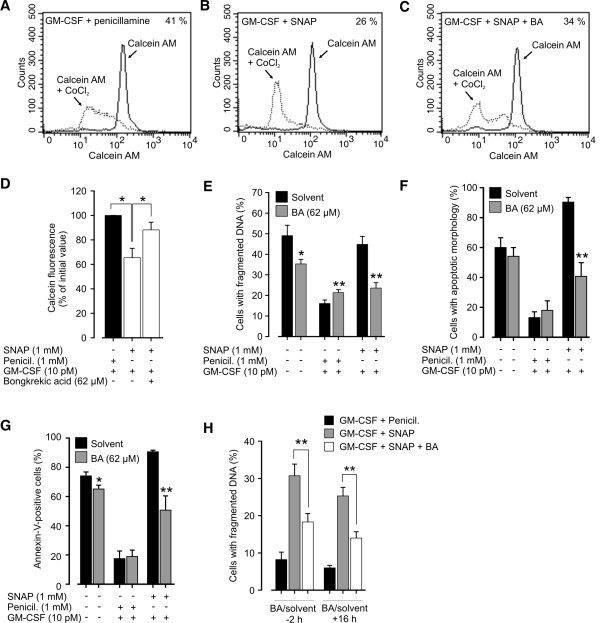
**SNAP-induced apoptosis is dependent on late mPT but preceded by early partial mPT.** Bongkrekic acid (62 μM) or solvent was added either 2 h before (**A-H**) or 16 h (**H**) after 10 pM GM-CSF, 1 mM SNAP and/or 1 mM penicillamine. In A-D Eosinophils were incubated for 1 h after which mPT was determined by calcein AM/CoCl_2_-method as described in materials and methods. In A-C shown are representative graphs from analysis of mPT and in the top right corner percentage of calcein fluorescence of its initial value describing the extent of mPT. In E-H total incubation time of eosinophils was 40 h after which apoptosis was determined by DNA fragmentation assay (**E**, **H**), morphological analysis (**F**) or Annexin-V FITC staining (**G**). Values are mean ± SEM from five to six independent experiments, * indicates p < 0.05 and ** p < 0.01 as compared with the respective control.

Mitochondrial membrane permeabilization including mPT and loss of ΔΨ_m_ are critical steps in mitochondrial apoptotic pathway [16]. We used calcein AM/CoCl_2_-method to assess whether SNAP induces mPT in GM-CSF-treated eosinophils. We found that SNAP reduced calcein fluorescence by 34.6 ± 7.7% when compared to penicillamine-treated cells at 1 h indicating that mPT occurs and CoCl_2_ enters mitochondria to quench calcein fluorescence (Figures 2A-D). This effect was mostly prevented by bongkrekic acid, an inhibitor of mPT (Figures 2A-D). However, when compared to ionomycin-induced complete mPT as indicated by total quench of calcein fluorescence (data not shown), mPT raised by SNAP was partial.

To determine whether mPT is important for apoptosis in the presence or absence of SNAP and GM-CSF, we treated the cells with bongkrekic acid. Indeed, bongkrekic acid significantly reversed the effect of SNAP on DNA fragmentation (Figure 2E), cell morphology (Figure 2F) and Annexin-V labeling (Figure 2G). Only a small portion of spontaneous apoptosis was prevented by treatment with bongkrekic acid (Figures 2E-G). To examine whether the early mPT at 1 h time-point is critical for SNAP-induced apoptosis, we added bongkrekic acid to eosinophils at later, arbitrary time-point, 16 h after SNAP and GM-CSF. Surprisingly, SNAP-induced apoptosis was prevented even when bongkrekic acid was added to the cells 16 h after SNAP and GM-CSF (Figure 2H) indicating that early partial mPT is not significant for apoptosis. Thus, the threshold for apoptosis-inducing complete mPT is achieved 16–40 h after addition of SNAP.

### SNAP induces late loss of mitochondrial membrane potential

**Figure 3:**
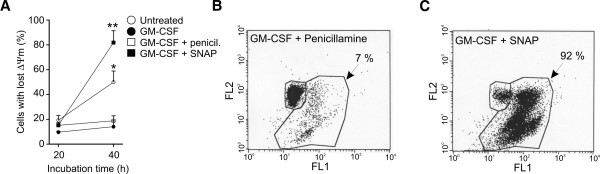
**SNAP induces late loss of ΔΨ**_**m**_**.** Eosinophils were incubated with solvent, SNAP (1 mM), penicillamine (1 mM) and/or GM-CSF (10 pM) for indicated times (**A**) or for 40 hours (**B**-**C**) after which JC-1-staining and analysis of mitochondrial membrane potential by flow cytometer was conducted as described in materials and methods. In A shown are mean ± SEM from five independent experiments. * indicates p < 0.05 and ** p < 0.01 when compared to cells treated with GM-CSF or GM-CSF and penicillamine, respectively. In B-C shown are representative graphs from analysis of ΔΨ_m_ with percentages of cells with lost ΔΨ_m_.

Two forms of mPT have been previously identified. Sustained opening of the mPT pore leads to loss of ΔΨm resulting in cell death. However, transient or flickering openings of the mPT pore may only lead to mitochondrial membrane depolarization spikes, but not to permanent loss of ΔΨ_m_ and cell death [21,22]. Next our aim was to determine the effect of SNAP on ΔΨ_m_ at different time-points to get further evidence that only mPT occurring at late stage results in loss of ΔΨ_m_ and is critical for apoptosis. As expected, we found that SNAP did not significantly increase the proportion of cells with lost ΔΨ_m_ after 20 h of incubation (Figure 3A). At 40 h time-point, loss of ΔΨ_m_ occurred in most of SNAP-treated but not penicillamine-treated cells (Figures 3A-C). This result supports the conclusion that SNAP induces permanent mPT leading to loss of ΔΨ_m_ and apoptosis at late stage.

### JNK mediates SNAP-induced apoptosis but is also induced by early non-apoptotic mPT

**Figure 4:**
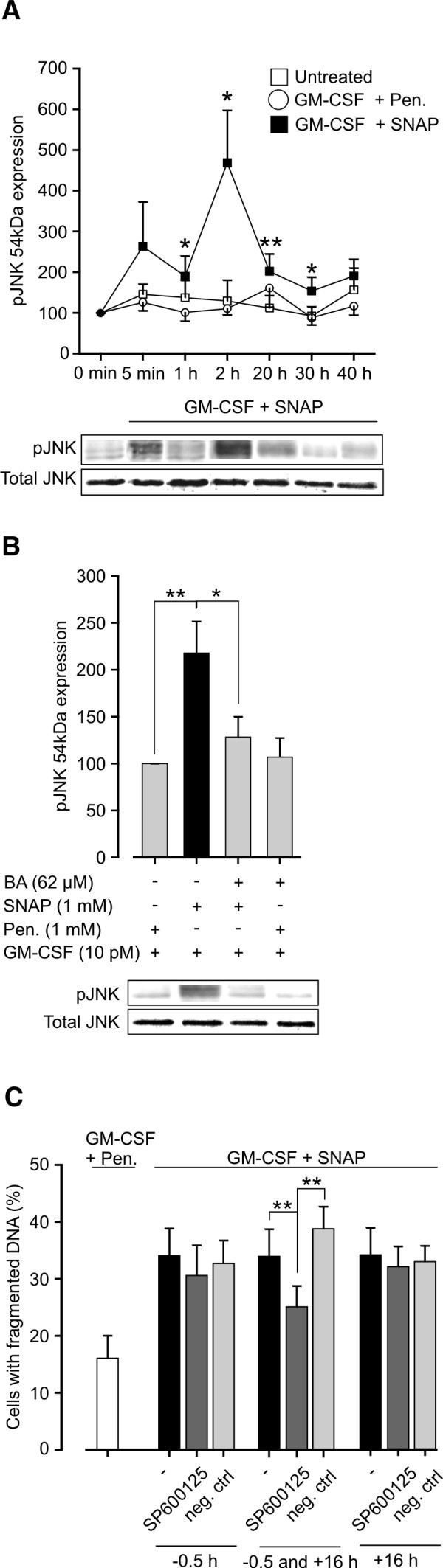
**JNK mediates SNAP-induced apoptosis but is also strongly induced by early non-apoptotic mPT.** In **A** eosinophils were incubated in the presence or absence of 1 mM SNAP, 1 mM penicillamine and/or 10 pM GM-CSF for indicated times and lysed for western blotting. pJNK and total JNK levels were normalized against 0 min GM-CSF (10 pM) sample. Total JNK was used as loading control and shown are 54 kDa pJNK and total JNK. * indicates p < 0.05 and ** p < 0.01 as compared with eosinophils stimulated with GM-CSF and penicillamine for the corresponding time, n = 7. Untreated cells were compared to 0 min GM-CSF sample, n = 5. In **B**, eosinophils were preincubated with bongkrekic acid for 2 h before adding SNAP, penicillamine and/or GM-CSF. Incubation was continued for 2 h before cell lysis and western blotting. Shown are mean ± SEM of 6 individual experiments. In **C**, time-points shown below the columns refer to the addition times of solvent, SP600125 (10 μM) or its negative control (10 μM). Time zero represents the time of adding GM-CSF (10 pM) and SNAP (1 mM) or penicillamine (1 mM). Total incubation time was 40 h and DNA fragmentation assay was used for determination of apoptosis. Values are mean ± SEM of 5 individual experiments. * indicates p < 0.05 and ** p < 0.01 versus respective control.

We have previously shown that SNAP-induced apoptosis is dependent on JNK and demonstrated early activation of JNK by SNAP [11]. To more carefully explore the pattern of JNK activation we studied pJNK levels during longer time-scale. SNAP induced a strong JNK phosphorylation at 2 h time-point and smaller increases in pJNK levels at 1 h, 20 h and 30 h time-points (Figure 4A). As expected, untreated eosinophils showed stable pJNK levels (Figure 4A). To examine if early activation of JNK is a consequence of early mPT, we used bongkrekic acid to inhibit mPT. Treatment with bongkrekic acid completely prevented SNAP-induced increase in pJNK levels at 2 h time-point (Figure 4B).

In previous studies it has been demonstrated that rapid, strong and transient JNK activation is a stress response resulting in cell survival signalling while delayed and sustained JNK activation is related to apoptosis [23,24]. To clarify whether the early (2 h) and/or late (20–30 h) JNK activation is important for SNAP-induced eosinophil apoptosis, we used JNK inhibitor SP600125 added before and/or 16 h after SNAP. We found that if SP600125 was added only at 16 h time-point it had no effect on SNAP-induced apoptosis (Figure 4C). Surprisingly, SP600125 added only before SNAP was not effective either (Figure 4C). Nevertheless, a clear inhibition of SNAP-induced apoptosis was seen when SP600125 was added at both of these time-points (Figure 4C). A chemically different inhibitor of JNK, JNK inhibitor VIII (1 μM), partly prevented SNAP-induced eosinophil apoptosis when added once 30 min before SNAP (P < 0.05, n = 7, data not shown). The results suggest that the later JNK activation is involved in mediating apoptosis but the later phase may begin earlier than 16 h and persist at least up to 30 h after addition of SNAP.

Activation of JNK has been previously shown to mediate cell death by participating in the induction of mPT and loss of ΔΨ_m_[25,26]. We did not find, however, any role for JNK in stimulating these mitochondrial changes because inhibition of JNK by SP600125 or JNK inhibitor VIII did not reverse SNAP-induced loss of ΔΨ_m_ at 40 h time-point (p > 0.05, n = 4-5, data not shown).

### Significant role of early ROS production in SNAP-induced apoptosis

**Figure 5:**
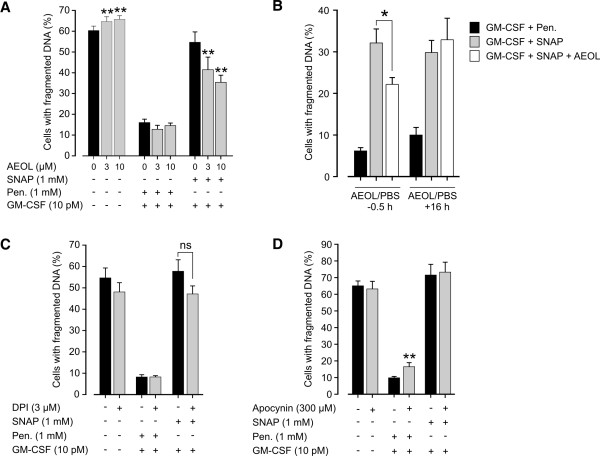
**Early ROS-production is involved in SNAP-induced apoptosis.** Eosinophils were incubated with AEOL for 15 min (**A**), DPI (**C**) or apocynin (**D**) for 20 min prior to stimulation with SNAP, penicillamine and/or GM-CSF for 40 hours. In **B**, AEOL was added 30 min prior to or 16 h after SNAP and GM-CSF. Apoptosis was measured by DNA fragmentation assay. * indicates p < 0.05 and ** p < 0.01 versus respective control. Shown are results from five to seven independent experiments.

Cellular stress often results in increased production of ROS such as superoxide (O_2_^-^), hydroxyl radical (·OH) and hydrogen peroxide (H_2_O_2_) by mitochondrial respiratory chain, NADPH oxidase or other enzymes such as cyclooxygenase (COX). These radicals participate in the generation of several toxic metabolites. One of the most toxic metabolites formed in the presence of superoxide and nitric oxide is peroxynitrite, which induces DNA damage, lipid peroxidation and inhibits several cytoplasmic and mitochondrial enzymes [27]. To determine whether SNAP-induced apoptosis in the presence of GM-CSF is dependent on superoxide and/or peroxynitrite formation, we used a small-molecule antioxidant AEOL 10150 with a structure analogous to the catalytic site of SOD [27]. AEOL partly prevented the pro-apoptotic effect of SNAP but not spontaneous apoptosis indicating that treatment with SNAP specifically increases cellular ROS production (Figure 5A). When AEOL was added 16 hours after SNAP and GM-CSF, the treatment had no effect any more on SNAP-induced apoptosis (Figure 5B) indicating importance of early production of ROS. Inhibitors of NADPH oxidase, DPI and apocynin, had no significant effect on SNAP-induced apoptosis suggesting that NADPH oxidase is not the source of superoxide (Figures 5C-D). As a conclusion, early superoxide and/or peroxynitrite production is an important step in SNAP-induced apoptosis but superoxide does not originate from NADPH oxidase.

## Discussion

Nitric oxide is produced in high amounts in asthmatic lungs and has an important role as a regulator of lung inflammation. In this study, we found that NO prevents the survival-prolonging effect of GM-CSF in eosinophils by inducing apoptosis consistently with our previous reports [11,12]. We focused here to the cascade of events leading to NO-induced eosinophil apoptosis particularly concentrating on the role of mitochondria, ROS and JNK. We showed that NO-induced eosinophil apoptosis is dependent on early ROS production, JNK and late mPT. In addition, we found that NO induced an early partial mPT and mPT-dependent JNK activation but those events seemed not mediate NO-induced apoptosis detected at later time points.

Mitochondrial permeability transition pore is a channel in the inner mitochondrial membrane that is composed of several proteins in a complex manner. The molecular structure of the channel has not yet been resolved despite of numerous attempts and it has been postulated that the structure may vary depending on the cell type and/or the trigger. Inhibitor of mPT, bongkrekic acid, acts as a ligand to adenine nucleotide translocator (ANT), which is either a component or an important regulator of mPT [28,29]. ANT has been shown to act as a critical target in mitochondrial membrane permeabilization induced by NO and peroxynitrite [30], giving ground for usage of bongkrekic acid as an inhibitor of mPT in this study. Here, we also showed that bongkrekic acid inhibits mPT in eosinophils.

Our results demonstrate that NO has a marked and varied effect on mPT at long time-scale. We found that NO induces partial mPT at 1 h in eosinophils that does not lead to early permanent loss of ΔΨ_m_. This strongly suggests that early NO-induced mPT is transient or flickering. Previously, flickering mPT has been demonstrated in healthy intact cells [21,31] and it has been postulated to act as a fast calcium release mechanism [32,33] participating thereby in diverse range of Ca^2+^ -mediated cellular activities. Flickering mPT may also be involved in cell protection during minor stress [34] or it may act as an early signal for oxidative stress-induced apoptosis [31]. In accordance with our results, NO was shown to induce and modulate mPT in a reversible manner in isolated mitochondria [35]. We found that the early mPT is not significant for initiation of NO-induced apoptosis because addition of mPT inhibitor bongkrekic acid at 16 h after SNAP and GM-CSF still efficiently prevented NO-induced apoptosis. This seems to be in contrast to the findings of Ma et al. [31] who showed that early flickering mPT and associated superoxide flashes induced by selenite are early signals of apoptosis. They showed that manipulation of selenite-induced flickering mPT and associated superoxide flashes by knockdown or overexpression of mPT component cyclophilin D decreased or increased selenite-induced apoptosis, respectively [31]. However, this manipulation also affects the irreversible mPT and there is no conclusive evidence that flickering mPT and the associated superoxide flashes are necessary for apoptosis. Nevertheless, because treatment with NO ends up in mPT-mediated apoptosis it is likely that flickering mPT is an important point where anti-apoptotic and pro-apoptotic signals converge and fate of the cell is determined. Most probably, if a certain threshold is achieved flickering mPT is turned into permanent mPT and cell undergoes apoptosis or necrosis.

We showed that the early partial mPT led to strong activation of JNK at 2 h. A smaller activation of JNK was observed at later time-points (20–30 h). Consistently, a previous study showed that apoptosis-inducing N-methyl-4-phenylpyridinium (MPP+) induced early and late phases of JNK activation in a mammalian cell line and the early phase was preventable by bongkrekic acid [36]. They also found that the late JNK activation was independent of mPT, which remains unsolved in our study. However, in contrast to our study, Casarino et al. did not show whether the early mPT-dependent JNK activation or late phase of JNK activation are relevant for MPP+ −induced apoptosis. To our knowledge, this is the first study to demonstrate mPT-mediated JNK activation not related to apoptosis. According to our study, one physiological function of flickering mPT may, therefore, be initiation of JNK signalling in response to oxidative stress probably aiming to cell rescue. Plenty of evidence from studies conducted by others supports the conclusion that the early mPT and the following JNK activation are a protective response. First, several groups have demonstrated that rapid, strong and transient JNK activation is a stress response resulting in cell survival signalling while delayed and sustained JNK activation is related to apoptosis [23,24]. Second, flickering mPT was shown to be involved in cell protection during minor stress [34]. Additionally, Beltran et al. have demonstrated that long exposure to NO by DETA-NONOate initially stimulates a protective response by inhibiting complex IV in the mitochondrial respiratory chain. This led to maintenance of ΔΨ_m_ by hydrolysis of glycolytic ATP instead of the respiratory chain and increased cell viability. The protective response induced by NO turned into apoptotic response by an unknown mechanism that was speculated to involve accumulation of oxidative damage [37]. In eosinophils ΔΨ_m_ is maintained exceptionally by hydrolysis of glycolytic ATP rather than respiratory chain in contrast to most eukaryotic cells [38] indicating that this mechanism is already functional in eosinophils. Evidence of the survival-increasing potential of NO in eosinophils has been shown by several groups [39-41]. Hebestreit and co-workers showed that NO stimulates eosinophil survival in the presence of apoptosis-inducing Fas at 24 h time-point [39]. Further time-points were not studied to see whether the survival signalling would have turned into apoptotic signalling. In our experiments GM-CSF produced maximal survival of eosinophils at 10 pM concentration making it impossible to show that NO activates survival machinery in eosinophils at early time-points. This evidence supports the conclusion that NO-induced early mPT and the following JNK activation are a protective response of the cell.

ROS/RNS are known inducers of JNK in eosinophils [42], which suggests that ROS formation may be involved in the early mPT-dependent activation of JNK. Studies by Zorov et al. [43] have shown a relationship between ROS and mPT which raises an interesting possibility for the mechanism of mPT-induced JNK activation. They showed that mitochondrial ROS accumulation leads to mPT, which was followed by mitochondrial ROS burst that can be released to the cytosol [43]. Also Ma et al. demonstrated mPT-dependent superoxide flashes in response to oxidative stress [31]. However, whether this mechanism explains mPT-mediated early JNK activation in NO-treated eosinophils remains to be clarified.

Stimulation of GM-CSF-treated eosinophils with NO resulted in permanent loss of ΔΨm and apoptosis at 40 h. Lost ΔΨ_m_ is often, but not always, a consequence of permanent mPT [44]. Prevention of NO-mediated apoptosis by late addition of bongkrekic acid, however, gives further evidence that the threshold for permanent mPT is crossed at time-point beyond 16 h. Previously, NO was shown to induce permanent mPT in isolated mitochondria and thymocytes [15]. In eosinophils, mPT mediated dexamethasone-induced apoptosis [17].

JNK has been previously reported to mediate spontaneous apoptosis and apoptosis induced by several drugs and nitric oxide in eosinophils [11,42,45]. In concordance with our previous results, we found here that NO activates JNK and JNK has a role in NO-induced apoptosis [11]. Similarly to the results with JNK peptide inhibitor 1 (L-JNKI1) in the previous study [11], two additions of JNK inhibitor SP600125 (at 30 min before and 16 h after SNAP) was required to suppress the pro-apoptotic effect of NO. Either addition alone had no statistically significant effect on SNAP-induced apoptosis. The result implies that the later phase of JNK activation is required for apoptosis but the later phase may initiate before 16 h. This is possible because we did not study pJNK levels in time-points between 2 h and 20 h. With the limited amounts of cells available for these studies and the sensitivity of the current assays, it was not possible to determine the exact time, when the later phase of JNK activation was initiated. The result also suggests that the initial 10 μM concentration of SP600125 was not adequate to inhibit JNK during long time-scale of activation in contrast to 1 μM concentration of JNK inhibitor VIII that slightly but statistically significantly suppressed the pro-apoptotic effect of SNAP. The mechanism by which JNK participates in NO-induced eosinophil apoptosis remains unclear. In previous studies with other cell types and stimulants JNK has promoted apoptosis by inducing mPT [25,26]. However, this seems not to be the mechanism in NO-induced apoptosis in eosinophils since inhibition of JNK had no effect on the SNAP-induced loss of ΔΨ_m_. It is also possible that JNK has a pro-apoptotic mechanism that is independent on mPT. For example, JNK-mediated transcription has been shown to enhance expression of Fas-ligand [46] indicating that JNK activation may stimulate extrinsic Fas pathway of apoptosis in parallel to intrinsic mitochondrial pathway. Alternatively, JNK-dependent mechanisms may mediate the pathway from mPT to DNA fragmentation.

Under normal physiological condition, few per cent of the oxygen consumed by mitochondria is converted to superoxide. Cellular stress often leads to further increase in superoxide production. Inhibition of the components of the mitochondrial respiratory chain has been demonstrated as one mechanism by which NO increases superoxide production [47]. Reaction between nitric oxide and superoxide leads to formation of peroxynitrite. By using SOD mimetic AEOL 10150, we showed that the pro-apoptotic effect of SNAP on eosinophils in the presence of GM-CSF is partly dependent on superoxide and/or peroxynitrite production. However, addition of SOD mimetic at 16 h time-point was no longer effective in reversing the pro-apoptotic effect of SNAP suggesting that early formation of superoxide and/or peroxynitrite is critical for SNAP-induced apoptosis. Early NO-induced ROS-production is in concordance with the results of other studies [48,49]. Previously in other cell types the peak of ROS production by NO has been demonstrated to occur before caspase activation and the following apoptosis [49,50]. However, the importance of this early ROS peak for apoptosis has been unclear. Our study shows that early increase of ROS is a critical event mediating NO-induced eosinophil apoptosis. NO-induced apoptosis seems to be initiated relatively late suggesting importance of accumulation of oxidative damage. Interestingly, because both apoptosis-related formation of ROS and activation of JNK seem to initiate before 16 h and ROS/RNS are known inducers of JNK [42], ROS may also participate in activating the later apoptosis-related JNK. Inhibition of apoptosis by AEOL was only partial suggesting that NO-induced formation of ROS is not the only key event for initiation of apoptosis. NO may also have direct apoptosis-stimulating effects on eosinophils. Another possibility that may explain the result is inefficiency of the used SOD-mimetic in dismutating all superoxide and peroxynitrite production. According to our results with inhibitors of NADPH oxidase, this enzyme is not the major source of superoxide in NO-treated eosinophils. This leaves mitochondrial electron transport chain as the most likely source of superoxide.

Nitric oxide is abundant in the lungs of asthmatics and thereby most likely affects eosinophil functions in a physiological situation. In this study, in addition to the mechanism of NO-induced apoptosis, we showed some interesting early events in NO-stimulated eosinophils that may take place even if the threshold for irreversible mPT and apoptosis is not crossed. In fact, levels of NO in the exhaled air has been shown to correlate to eosinophilic inflammation [8,9] which makes it tempting to speculate that NO might only induce flickering mPT and JNK activation ending up in a protective response but not apoptosis in a physiological situation.

## Conclusions

**Figure 6:**
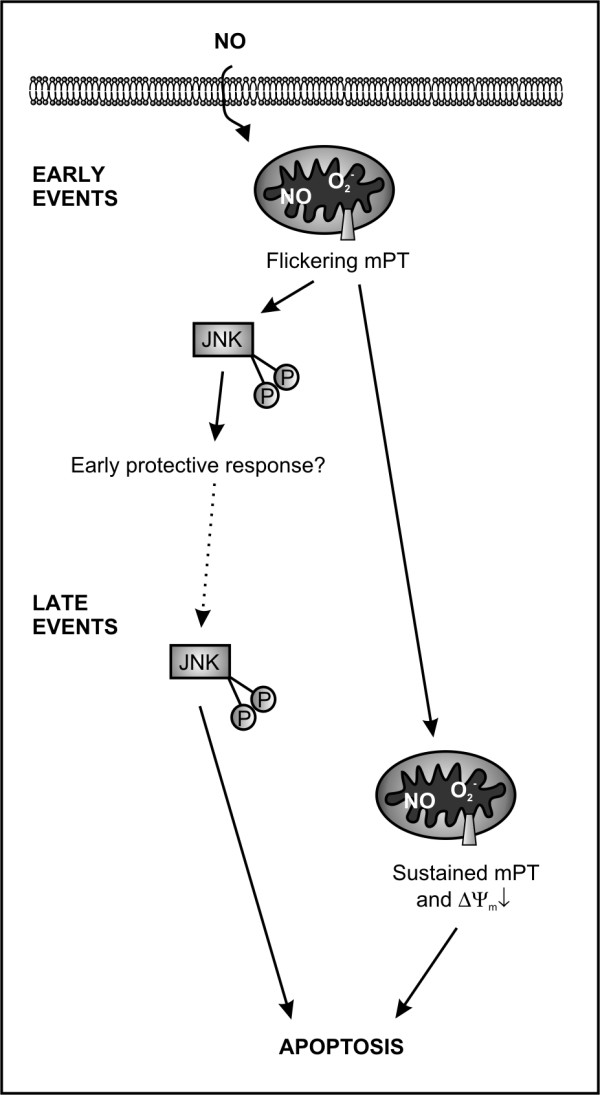
**A proposed model of the action of NO in GM-CSF-treated eosinophils.** As early events (1–2 hours), NO induces flickering mPT that leads to JNK activation but these events are not related to apoptosis and may act as an early protective response. Later, the flickering mPT turns into sustained mPT, mitochondrial membrane potential (ΔΨ_m_) collapses and cell undergoes apoptosis. A new phase of JNK activation occurs before late loss of mitochondrial function and is involved in mediating apoptosis but not via inducing disruption of mitochondrial function.

We showed here that NO induces apoptosis of GM-CSF-treated human eosinophils by a mechanism that involves early oxidative stress, JNK activation and late mPT. However, before irreversible activation of apoptotic machinery NO induces early flickering mPT that leads to JNK activation but is not related to apoptosis (Figure 6). Thereby, we showed some interesting early events in NO-stimulated eosinophils that may take place even if the threshold for irreversible mPT and apoptosis is not crossed. This study also revealed a previously unknown physiological function for transient mPT by showing that it may act as an initiator of non-apoptotic cell signal transduction by activating JNK.

## Competing interests

The authors declare that they have no competing interests.

## Authors’ contributions

PI-S participated in the study design, carried out experiments, performed data analysis and wrote the manuscript. EM participated in conceiving of the study and the study design and revised the manuscript critically for important intellectual content. VLK revised the manuscript critically for important intellectual content. HK conceived the study, participated in the study design, data analysis and contributed to the writing of the manuscript. All authors read and approved the final manuscript.
